# Mathematical Modeling of Local Drug Delivery in the Oral Cavity: From Release Kinetics to Mini-PBPK and Local PK/PD with Applications to Periodontal Therapies

**DOI:** 10.3390/pharmaceutics18010101

**Published:** 2026-01-12

**Authors:** Rafał Rakoczy, Monika Machoy-Rakoczy, Izabela Gutowska

**Affiliations:** 1 Department of Chemical and Process Engineering, Faculty of Chemical Technology and Engineering, West Pomeranian University of Technology in Szczecin, 70-310 Szczecin, Poland; 2 Department of Periodontology, Pomeranian Medical University, 70-111 Szczecin, Poland; 3 Department of Medical Chemistry, Pomeranian Medical University, 70-111 Szczecin, Poland

**Keywords:** mini-PBPK, drug release kinetics, subgingival drug delivery

## Abstract

Background/Objectives: Mathematical modelling provides a quantitative way to describe the fate and action of drugs in the oral cavity, where transport processes are shaped by salivary flow, pellicle formation, biofilm structure and the wash-out effect of gingival crevicular fluid (GCF). Local pharmacokinetics in the mouth differ substantially from systemic models, and therefore a dedicated framework is required. The aim of this work was to present a structured, physiologically based concept that links in vitro release testing with local pharmacokinetics and pharmacodynamics. Methods: A narrative review with elements of systematic search was conducted in PubMed, Scopus and Web of Science (1980–2025) for publications describing drug release, local PBPK, and PK/PD modelling in the oral cavity. Mathematical formulations were grouped into release kinetics, mini-PBPK transport and local PK/PD relations. Classical models (Higuchi, Korsmeyer–Peppas, Peppas–Sahlin) were integrated with a mini-PBPK structure describing saliva–mucosa–biofilm–pocket interactions. Results: The combined model captures adsorption to pellicle, diffusion within biofilm and wash-out by GCF. It allows simulation of variable clinical conditions, such as inflammation-related changes in Q_GCF_, and links local exposure to pharmacodynamic outcomes. Case studies with PerioChip^®^, Arestin^®^, and Atridox^®^ demonstrate how mechanistic models explain observed therapeutic duration and low-systemic exposure. Conclusions: The proposed mini-PBPK framework bridges empirical release data and physiological transport in the oral cavity. It supports rational formulation design, optimisation of local dosage, and personalised prediction of drug retention in gingival pockets. This modelling approach can become a practical tool for the development of dental biomaterials and subgingival therapies.

## 1. Introduction

Periodontal diseases remain widespread, and although scaling and root planing (SRP) form the foundation of treatment, many clinical situations benefit from the adjunctive use of locally delivered pharmacotherapy [1,2,3]. Local administration offers a practical way to achieve therapeutic concentrations in the periodontal pocket while keeping systemic exposure low. In this setting, the combination of inflammation, ulcerated epithelium and deep anaerobic niches creates transport conditions that differ markedly from those in other mucosal tissues, making quantitative modelling a useful complement to clinical observation [2,4].

The oral cavity is a highly dynamic environment. Saliva is continuously renewed, buffering local pH and influencing both ionisation and wash-out of active substances [3]. The pellicle and biofilm form interfacial layers that bind drug molecules and slow their movement, while gingival crevicular fluid (GCF) provides a steady outflow from the pocket [4]. GCF flow increases substantially during inflammation—often approaching an order-of-magnitude increase compared with health—and this variability has direct implications for local drug retention and clearance [4,5]. Recent clinical findings also show that periodontal inflammation and its impact on patient-reported outcomes vary considerably between individuals, even in those with well-controlled systemic diseases, highlighting the importance of accounting for inter-individual variability when modelling local drug exposure in GCF [6].

Over the past decade, a wide range of delivery systems have been introduced into periodontology, including biodegradable chips, microspheres, in situ forming gels and bioadhesive films [1,2,3]. Their clinical performance depends not only on the drug they carry but also on how the material releases it and how the oral environment distributes it. To capture this sequence of processes, we organize the modelling tools used in this work along a practical “carrier-to-effect” continuum that combines classical release models, a mini-PBPK structure tailored to the oral cavity, and local PK/PD indices such as T>MIC, AUC/MIC and C_max_/MIC derived from concentrations in GCF and biofilm.

Recent years have seen increasing interest in mechanistic and physiologically informed modelling approaches for local drug delivery, particularly in complex biological environments where systemic assumptions are no longer sufficient. Contemporary studies highlight the need to integrate formulation-dependent release, tissue-specific transport, and local pharmacodynamic effects within unified predictive frameworks. Despite this progress, applications to the oral cavity remain fragmented, often focusing on isolated components such as release kinetics or antimicrobial efficacy without an explicit physiological context. The present work addresses this gap by proposing a mini-PBPK framework that explicitly links in vitro release data with oral transport processes and local PK/PD outcomes [7,8,9].

In this framework, we aim to show how physiological realism and computational tractability can be combined to support both the interpretation of existing formulations and the rational design of new subgingival therapies.

## 2. Materials and Methods

This review was conducted as a narrative synthesis enriched with structured elements to ensure transparency and reproducibility. The aim was to capture the breadth of mathematical and computational approaches applied to local drug delivery in the oral cavity, with particular emphasis on subgingival systems used in periodontology.

The primary literature search covered PubMed/MEDLINE, Scopus and Web of Science from 1980 to October 2025. These databases were selected because they provide broad coverage of pharmacological, dental and bioengineering research. To supplement this search, information from FDA and EMA repositories was reviewed, with a focus on product labels, modelling guidance and regulatory expectations related to PBPK analyses. Search terms combined pharmacological and dental concepts—such as oral cavity, gingival crevicular fluid, pellicle, biofilm, local delivery, drug release, PBPK, mathematical model and kinetics—and were applied using MeSH terms and Boolean operators.

Study selection followed predefined criteria. We included studies that developed or applied mathematical models describing drug release, transport, or pharmacokinetics/pharmacodynamics within oral tissues or fluids, as well as preclinical and clinical studies reporting quantitative measurements in GCF, saliva, or biofilm matrices. Regulatory and methodological publications relevant to local or mucosal PBPK modelling were also considered. Studies without quantitative components, systemic-only models unrelated to local delivery, and non-peer-reviewed reports were excluded.

Two reviewers independently screened titles and abstracts before assessing full texts. From each eligible study, we extracted information on model structure, equations, assumptions, parameters, validation methods and clinical or biological endpoints. Owing to substantial heterogeneity across modelling strategies, a formal meta-analysis was not performed. Instead, findings were grouped into three thematic domains: (1) release kinetics, (2) mini-PBPK and local transport, and (3) local PK/PD and validation.

Methodological quality was judged based on clarity of model description, justification of assumptions, origin and plausibility of parameter values, and evidence of validation or sensitivity testing. Where possible, reported equations and parameter sets were cross-referenced with experimental measurements from dental and pharmacological literature to ensure physiological relevance.

## 3. Modeling Tools in Dental Applications

Mathematical approaches originally developed for systemic pharmacokinetics have found increasing relevance in dental pharmacology, provided they are interpreted within the unique physiology of the oral cavity. Rather than viewing these tools as isolated formulas, they can be understood as complementary ways of describing what happens to a drug as it moves from a formulation into saliva, across tissues and biofilm, and ultimately into a periodontal pocket.

### 3.1. Compartmental Models (Classical PK)

Classical one- and two-compartment models describe drug disposition under the assumption of rapid mixing within each compartment. For illustrative purposes, an intravenous bolus is often represented as $C\left(t\right)=C_{0}\;e^{-kt}$, with clearance defined as CL=V⋅k. Oral dosing can be described using the Bateman or transit-compartment models, whereas situations in which absorption is slower than elimination may exhibit flip-flop kinetics (*kₐ* < *k*). In the context of locally delivered periodontal therapies, systemic exposure is typically low, yet these models provide a useful framework for interpreting any absorbed fraction and estimating its clearance.

### 3.2. Nonlinearities and Special Phenomena

Local concentrations in the mouth may reach levels at which elimination becomes saturable. Under such conditions, Michaelis–Menten kinetics $CL\left(C\right)=\frac{V_{max}}{K_{m}+C}$ [10,11].

More accurately reflects the relationship between dose and AUC. Strong, reversible binding to oral surfaces may also introduce nonlinear behavior resembling target-mediated drug disposition; quasi-steady-state or quasi-equilibrium approximations help simplify these dynamics without losing physiological meaning.

### 3.3. PBPK and Physiological Context

PBPK models map tissues, flows (*Qₜ*), volumes and partition coefficients (*Kₚ*) to create a mechanistic representation of drug distribution. When adapted to the oral cavity, such models distinguish perfusion-limited from permeability-limited regions and allow integration of in vitro and in silico data. Their strength lies in enabling physiological extrapolation—such as simulating how changes in salivary flow, mucosal permeability or GCF wash-out alter local exposure profiles.

### 3.4. Population Modelling and Parameter Estimation

Nonlinear mixed-effects (NLME) methods separate typical population values from inter-individual or inter-occasion variability. Diagnostic approaches—such as goodness-of-fit plots and prediction-corrected visual predictive checks (pcVPC or rcVPC)—help assess how well simulations reproduce observed variability. Given the substantial heterogeneity in salivary flow, inflammation and biofilm structure across patients, population-based approaches are essential for developing models that generalize to real clinical populations.

Diagnostic tools such as prediction-corrected visual predictive checks help determine whether simulated profiles encompass the range of observed patient responses. When the model reproduces both the central tendency and spread of the data, confidence increases that it can be used to explore clinical scenarios, compare formulations, or refine dosing strategies.

## 4. Mini-PBPK of the Oral Cavity: Saliva–Mucosa–Biofilm–Pocket

### 4.1. Assumptions and System Parameters

The oral cavity can be represented as a series of interconnected regions that together determine how a drug behaves after local administration. In the mini-PBPK structure, five elements are considered: saliva (*S*), the pellicle-covered mucosal surface (*M*), the underlying mucosal tissue (*T*), the dental biofilm (*B*) and the periodontal pocket (*P*). Each compartment reflects a distinct physiological role.

Saliva serves as the primary fluid medium, with rapid renewal and dilution capability. The pellicle and superficial mucosal layers create an interface where adsorption and early partitioning occur. Beneath this surface, mucosal tissue acts as a permeable barrier that can absorb a portion of the dose and contribute to local clearance. The dental biofilm introduces diffusional resistance due to its dense extracellular matrix, creating gradients that slow drug transport. Finally, the periodontal pocket behaves as a semi-isolated chamber in which a drug can accumulate but is steadily removed by gingival crevicular fluid (GCF).

Parameter values for these regions—such as volumes, flow rates, permeabilities and effective diffusion coefficients—are chosen based on physiologically plausible ranges and differ between resting and stimulated conditions. Importantly, GCF flow increases markedly during inflammation, reducing local residence time. Recognising this variability is essential for interpreting model predictions and applying them to individual clinical scenarios.

### 4.2. Structure and Equations

To complement the narrative description, we reintroduce the full system of equations together with typical physiological ranges used in simulation studies. These values serve as illustrative examples and are not intended to represent a specific clinical case.

(1) Saliva compartment (S)

$$\frac{dC_{S}}{dt}=\frac{1}{V_{S}}\left(R_{in}-Q_{S}C_{S}-J_{S\to M}\cdot A_{SM}-J_{S\to B}\cdot A_{SB}-J_{S\to P}\cdot A_{SP}\right)$$

Typical values: *V_S_* ≈ 0.7–1.0 mL; *Q_S_* (resting) ≈ 0.3–0.5 mL/min; *Q_S_* (stimulated) ≈ 1–2 mL/min.

A short numerical example: for chlorhexidine mouthrinse (C_S,0_ ≈ 2 mg/mL), resting saliva flow (0.4 mL/min) reduces salivary concentration by approximately 40% during the first minute purely by dilution.

(2) Pellicle/Mucosa (M)—adsorption

$$\frac{d\Gamma }{dt}=k_{\mathrm{ads}}\;C_{S}\;\left(\Gamma_{max}-\Gamma \right)-k_{\mathrm{des}}\;$$

Example values: Γ_max_ ≈ 5–15 µg/cm^2^; *k_ads_* ≈ 0.1–1.2 mL·cm^−2^·min^−1^; *k_des_* ≈ 0.01–0.1 min^−1^.

This behaviour explains why chlorhexidine retains activity long after salivary levels decline.

(3) Interfacial flux S→M

$$J_{S\to M}=P_{SM}\left(C_{S}-\frac{C_{T}}{K_{p,T}}\right)$$

Typical: *P_SM_* ≈ 10^−5^–10^−4^ cm/min; *Kp, ST* ≈ 2–10.

(4) Mucosal tissue (T)

$$\frac{dC_{T}}{dt}=\frac{P_{SM}A_{SM}}{V_{T}}\left(C_{S}-\frac{C_{T}}{K_{p,T}}\right)-\frac{CL_{\mathrm{local}}}{V_{T}}C_{T}$$

Example values: *V_T_* ≈ 0.05–0.1 mL; *A_SM_* ≈ 2–6 cm^2^; *CL_local_* ≈ 0.01–0.1 mL/min.

(5) Biofilm diffusion (1-D PDE)

$$\frac{\partial C_{B}}{\partial t}=D_{\mathrm{eff}}\frac{\partial^{2}C_{B}}{\partial x^{2}}-k_{\mathrm{bind}}\;C_{B}$$

(6) Boundary conditions

$$C_{B}\left(0,t\right)=C_{S}\left(t\right),\;{\frac{\partial C_{B}}{\partial x}|}_{x=L_{B}}=0,\;J_{B\to P}\left(t\right)=-\;D_{\mathrm{eff}}{\frac{\partial C_{B}}{\partial x}|}_{x=L_{B}}$$

Typical values: *D_eff_* ≈ 1 × 10^−7^–5 × 10^−7^ cm^2^/s; *L_B_* ≈ 100–300 µm.

Illustration: for doxycycline (Deff ≈ 3 × 10^−7^ cm^2^/s), the characteristic diffusion time across a 200 µm biofilm is t ≈ *L^2^/D* ≈ (0.02 cm)^2^/3 × 10^−7^ ≈ 1300 s (~22 min).

(7) Gingival pocket (P)

$$\frac{dC_{P}}{dt}=\frac{J_{B\to P}A_{BP}+R_{\mathrm{release}}\left(t\right)-Q_{\mathrm{GCF}}\;C_{P}}{V_{P}}$$

Typical clinical values: *V_P_* ≈ 0.5–2 µL; *Q*_GCF_ (healthy) ≈ 0.05–0.1 µL/min; *Q*_GCF_ (inflamed) ≈ 0.2–0.5 µL/min.

Numerical example for PerioChip^®^: with V_P_ = 1 µL and *Q*_GCF_ = 0.3 µL/min, the natural clearance half-life in the pocket is t½ ≈ (V_P_ ln2)/ *Q*_GCF_ ≈ (1 × 0.693)/0.3 ≈ 2.3 min. This demonstrates why binding and slow release are essential to maintain CHX levels.

To translate the physiology of the oral cavity into a computational framework, the model relies on a set of interconnected mass-balance equations. Rather than treating these equations as isolated mathematical objects, they can be understood as a narrative of how a drug moves through the oral environment—from initial release, through interfaces and tissues, and finally into the periodontal pocket.

The saliva compartment serves as the point of entry for most locally delivered formulations. Its behavior is governed by a balance between input from the drug carrier, dilution by saliva flow, and transfer toward adjacent regions. Because saliva is refreshed rapidly, even modest changes in flow can substantially influence the early concentration profile. This dynamic makes the saliva compartment an important driver of early exposure.

At the mucosal surface, the pellicle acts not simply as a passive boundary but as an active layer capable of binding and releasing drug molecules. The adsorption term captures how a portion of the dose becomes temporarily stored in this layer, while desorption allows that stored fraction to re-enter the fluid phase. This reversible exchange is notably important for agents like chlorhexidine, which exhibit strong affinity for oral surfaces.

Beneath the pellicle, the mucosal tissue forms a permeable barrier through which the drug may diffuse before undergoing local clearance. This step is represented by a second mass-balance equation in which permeability, surface area and tissue volume determine how much of the drug crosses into the tissue. Such movement is generally modest for hydrophilic antimicrobials used in periodontal therapy but becomes more relevant for agents with systemic absorption potential.

The biofilm compartment introduces spatial structure into the model. Here, a diffusion–reaction equation reflects the dense extracellular matrix that slows molecular movement and the possibility of reversible binding within the biofilm. Although simplified to one dimension for computational tractability, this representation captures the essential feature of spatial gradients, with higher concentrations near the saliva interface and progressively lower levels toward the pocket.

Finally, the periodontal pocket integrates influx from the biofilm with any direct release from subgingivally placed formulations. Wash-out through the gingival crevicular fluid forms the main elimination pathway in this region, and its rate strongly depends on the degree of inflammation. By combining these processes, the pocket equation estimates how long therapeutic concentrations persist at the intended site.

Together, these linked equations form a cohesive description of drug behavior in the oral cavity. Each component represents a physiological process, and the interactions among them determine whether a formulation achieves sustained local exposure. Understanding these relationships is essential for interpreting simulations and for guiding the design of more effective local therapies.

### 4.3. Starting Ranges and Identifiability

Selecting appropriate parameter ranges is crucial for generating reliable simulations. Several physiological processes—mucosal permeability, biofilm diffusion and pellicle adsorption—can produce similar effects on observable concentrations. Without anchoring parameters in independent measurements, models may suffer from compensation, where one parameter masks inaccuracies in another.

To avoid this, parameter selection is guided by laboratory methods: adsorption constants (*k*_ads_, *k*_des_, Γ_max_) may be informed by QCM-D studies; effective diffusion (*D_eff_*) by FRAP or confocal microscopy; and transmucosal permeability (*P_SM_*) by Ussing-chamber experiments. Using such experimentally derived anchor points improves physiological credibility and reduces uncertainty in downstream simulations.

Identifiability also plays a key role. Some parameters can be estimated from clinical data, while others should remain fixed. GCF flow, in particular, varies substantially with inflammation and must be calibrated to the patient’s status rather than assumed from averaged values.

Parameters obtained from in vitro techniques such as QCM-D or FRAP are not transferred directly to the in vivo mini-PBPK model but are instead used as anchor points defining physiologically plausible parameter ranges. Adsorption constants measured on pellicle-coated sensors provide upper-bound estimates for *k*_ads_, which are subsequently adjusted to account for surface heterogeneity, salivary flow, and the dynamic turnover of the oral mucosa. Similarly, effective diffusion coefficients obtained from biofilm-mimicking systems are interpreted as reference values and scaled by considering in vivo biofilm thickness, porosity, and hydration state. This pragmatic, range-based parameterization approach acknowledges experimental uncertainty while preserving physiological realism and reproducibility of the model.

### 4.4. Clinical Mapping

The mini-PBPK framework helps explain why different local delivery systems behave differently in vivo. For chlorhexidine rinses or PerioChip^®^ [12], an initial burst of drug into saliva is often followed by prolonged retention due to strong pellicle binding. Transmucosal dosage forms, such as oral dissolving films, partition part of the dose through the mucosa while another fraction is swallowed, requiring explicit representation of both local and systemic pathways.

By aligning each formulation with the physiological processes it engages—salivary dilution, surface adsorption, mucosal permeation, biofilm diffusion, or direct deposition into the pocket—the model contextualizes observed concentration–time profiles and provides a basis for comparing therapeutic strategies in different clinical scenarios.

## 5. Release from Biomaterials and Integration into the Pocket (P)

### 5.1. Release Models

Local drug delivery systems used in periodontology rely on distinct release mechanisms, and choosing an appropriate mathematical model helps link in vitro profiles with in vivo performance. Diffusion-controlled systems are often described by the Higuchi equation (Mt/M∞ = kH·t^1/2^), whereas the Korsmeyer–Peppas model (Mt/M∞ = k·tⁿ) allows the exponent *n* to distinguish between Fickian, anomalous, or relaxation-driven release. Mixed-mechanism systems such as PLGA microspheres may be captured with the Peppas–Sahlin model (F = k_1_·tᵐ + k_2_·t^2^ᵐ), and geometrical changes during dissolution or erosion are frequently represented by the Hixson–Crowell relation (W_0_^1^ᐟ^3^ − Wt^1^ᐟ^3^ = kHC·t). For spherical formulations, the Baker–Lonsdale model provides a more realistic description, while the Weibull function offers a flexible empirical option for complex or multi-phase release profiles [13,14,15,16,17].

Selecting among these models depends on identifying the dominant physical processes under oral conditions. Stress testing under varying pH, salivary flow, or ionic strength helps reveal whether diffusion, polymer relaxation, swelling, or erosion governs release. Objective comparison with AIC or BIC further supports model selection and prevents over-interpretation of noisy in vitro data.

### 5.2. Feeding R_release_(t) into C_p_(t)

Understanding how a formulation releases a drug in vitro addresses only part of the pharmacokinetic picture. To predict local exposure in a periodontal pocket, the release-rate function *R*_release_(t) must be coupled with physiological transport processes. The corresponding differential equation:

$$\frac{dC_{P}}{dt}=\frac{1}{V_{P}}\left(J_{B}PA_{B}P+R_{\mathrm{release}}\left(t\right)-Q_{GCF}C_{p}\right)$$

Combines three key influences:

– input from the biofilm interface,
– input from the formulation itself, and
– wash-out through GCF, which often dominates elimination in inflamed sites.

Pocket volume (*V_P_*) and GCF flow (*Q*_GCF_) vary substantially across patients, and small changes in either parameter can lead to pronounced differences in *C*_p_ (*t*). Therefore, scaling *R*_release_(t) to GCF dynamics is essential when translating in vitro release profiles into clinically meaningful predictions.

In this formulation, the two source terms represent distinct physical drug entry pathways. The term $J_{B}P\cdot A_{B}P$ describes drug transport from the biofilm into the periodontal pocket and is relevant for scenarios in which the biofilm acts as an intermediate diffusion barrier. In contrast, *R*_release_(t) represents a direct source term associated with subgingivally placed delivery systems (e.g., chips or in situ gels), where the formulation releases the drug directly into the pocket lumen, partially bypassing the biofilm. Depending on the clinical formulation and its placement, one of these terms may dominate, while the other can be negligible.

## 6. Case Studies (Subgingival Therapies)

Subgingival delivery systems differ substantially in how they release the drug, interact with oral structures and respond to local physiological conditions. The examples below illustrate how the mini-PBPK framework contextualizes these behaviors and explains observed clinical performance.

### 6.1. PerioChip^®^ (Chlorhexidine)

PerioChip^®^ provides a classic biphasic release pattern: an initial 24 h burst followed by a quasi–zero-order phase lasting 7–10 days. The strong affinity of chlorhexidine for pellicle and biofilm surfaces means that even modest early release results in prolonged retention. In simulations, this appears as a rapid rise in salivary concentration, followed by slow redistribution and clearance dominated by GCF. Local concentrations typically remain above the MIC range while systemic exposure is negligible [18,19,20].

### 6.2. Arestin^®^ (Minocycline Microspheres)

Arestin^®^ uses PLGA microspheres that release the drug through a combination of diffusion and polymer degradation. These kinetics are well represented by Peppas–Sahlin or Baker–Lonsdale models. Clinical performance depends strongly on inflammatory status: higher Q_GCF_ in inflamed pockets accelerates wash-out and shortens the duration of therapeutic levels. The model captures these interactions, illustrating why the same formulation may perform differently in pockets with different inflammatory burdens [1].

### 6.3. Atridox^®^ (Doxycycline In Situ Gel)

Atridox^®^ forms a semi-solid matrix after in situ gelation. The system provides sustained doxycycline release for approximately seven days, producing concentrations in GCF that exceed MIC_90_ while maintaining minimal plasma levels. Because the drug is deposited directly into the pocket, the influence of salivary dilution is limited; instead, local clearance depends mainly on Q_GCF_ and biofilm uptake [21].

Across these examples, the mini-PBPK structure demonstrates its flexibility. Despite involving distinct materials and release mechanisms, all three systems can be mapped onto the same physiological processes—adsorption, diffusion, retention and wash-out—providing a mechanistic basis for comparing local therapies and predicting patient-specific outcomes.

## 7. Linking Exposure and Effect (Local PK/PD)

Local PK/PD relationships allow drug concentrations in the periodontal pocket to be translated into microbiological and inflammatory effects. Classical antimicrobial indices such as T>MIC, AUC/MIC and *C_MAX_*/MIC can be derived from simulated concentration–time profiles and are closely linked with pharmacodynamic outcomes in infectious disease modelling [20].

For describing bacterial killing, *E_MAX_* or Hill-type models are commonly used to capture the saturable nature of antimicrobial effect [22]. Inflammatory biomarkers such as IL-1β or MMP-8 are more accurately represented by indirect-response models, where drug action inhibits mediator production or accelerates its clearance [19]. When the onset of effect is delayed relative to measured concentrations, an effect compartment defined by k_e0 can be introduced to account for hysteresis between exposure and response [23].

Recent clinical evidence also shows that difficulties in achieving rational antibiotic use in periodontal practice remain a relevant issue, underscoring the importance of antibiotic-sparing, locally delivered therapies supported by quantitative PK/PD modelling [5].

## 8. Study Design and Validation

Designing robust studies for oral drug delivery requires careful attention to the environments in which local concentrations are measured. Gingival crevicular fluid (GCF) changes rapidly during the initial post-dosing period, making dense sampling essential in the first hours, whereas later time points can be captured with daily measurements. Saliva should be collected under both resting and stimulated conditions because flow rate strongly influences dilution and transport [24]. Measurements in pellicle or biofilm layers benefit from surface-oriented techniques that detect adsorption and slow diffusion rather than bulk fluid levels [25].

A coherent analytical workflow begins with characterizing in vitro release profiles to obtain $R_{\mathrm{release}}\left(t\right)$, which then feeds into the local transport model and, when appropriate, into PK/PD components. Handling these steps in a structured sequence prevents inconsistencies between how a formulation releases drug, how it distributes across oral tissues, and how it ultimately produces biological effects [26].

Model performance is evaluated using diagnostic tools that reveal how well simulated profiles match observed data. Prediction-corrected visual predictive checks, residual analyses and sensitivity assessments help identify the parameters that most strongly influence system behavior [24]. External datasets—whether from independent studies, different patient groups, or alternative sampling strategies—provide valuable opportunities to test generalizability and strengthen model credibility [26]. Through this layered approach, the modelling framework becomes not only scientifically robust but also clinically informative.

## 9. Limitations

Several limitations of the present framework arise from the complexity of the oral environment. Representing the biofilm as a one-dimensional, homogeneous layer inevitably simplifies a structure that is far more heterogeneous in reality, with micro-channels, density gradients and regional differences in nutrient and oxygen availability influencing how drugs penetrate and bind within the matrix [24]. The oral cavity also exhibits substantial spatial variability in salivary flow and shear forces, yet these dynamics are approximated in the model by a single effective flow term.

Parameter interdependence introduces an additional challenge. Permeability (*P_SM_*), effective diffusion (*D_eff_*) and adsorption parameters (*k_ads_*, *k_des_*, Γ) may compensate for one another if not anchored to independent experimental measurements. Such compensation can produce apparently good fits while masking inaccuracies in the underlying physiology [26]. Laboratory assays, including QCM-D, FRAP/CLSM and Ussing-chamber studies, help constrain these parameters but cannot eliminate uncertainty entirely.

Gingival crevicular fluid flow (*Q_GCF_*) varies widely between individuals and increases substantially during inflammation. Because *Q_GCF_* strongly influences drug clearance from the pocket, simulations should be calibrated to the patient’s inflammatory status rather than relying on population averages [18]. Finally, biofilm-adjusted minimum inhibitory concentrations often differ markedly from planktonic MIC values, underscoring the need for caution when extrapolating systemic PK/PD thresholds to subgingival conditions [25].

Potential sources of independent data for external validation include published clinical studies reporting time-resolved drug concentrations in gingival crevicular fluid following local delivery (e.g., studies on PerioChip^®^ or Atridox^®^), as well as controlled clinical trials comparing different subgingival formulations under defined inflammatory conditions. Advanced experimental approaches, such as microdialysis or microperfusion measurements in gingival tissues, although technically demanding, would provide particularly valuable datasets for testing local concentration predictions. In addition, publicly available regulatory dossiers and post-marketing studies may serve as reference benchmarks for validating model-derived exposure profiles.

## 10. Discussion

Mathematical modelling has become an indispensable part of modern pharmaceutical sciences, yet its application in dentistry remains relatively limited. The oral cavity presents unique challenges: a constantly changing environment, dynamic salivary and GCF flow, and the coexistence of soft and hard tissues with markedly different permeabilities. Early empirical work on subgingival therapies focused on describing drug diffusion through polymeric films or gels, often relying on the Higuchi or Korsmeyer–Peppas models [1,2,3]. Although valuable, these in vitro approaches did not capture fluid turnover or adsorption dynamics. Later developments introduced compartmental methods linking in vitro release with concentration–time profiles in GCF or saliva, but anatomical detail remained limited.

The mini-PBPK model proposed here extends these foundations by incorporating adsorption–desorption at pellicle and mucosal surfaces, biofilm diffusion, and GCF-driven wash-out. This physiological structure explains why distinct formulations behave differently in vivo. For example, the biphasic profile of PerioChip^®^—marked by an initial burst followed by prolonged retention—reflects both material properties and strong surface binding [19]. Minocycline microspheres (Arestin^®^) illustrate how diffusion and polymer degradation together determine release, with clinical performance strongly influenced by inflammation-related increases in Q_GCF_ [24]. Atridox^®^, a doxycycline in situ gel, highlights how direct deposition into the pocket produces sustained concentrations with minimal systemic absorption [25].

These examples illustrate the broader value of mechanistic modelling. The ability to simulate “what-if” clinical scenarios—such as pockets with elevated Q_GCF_ or altered biofilm density—allows hypotheses to be tested that would be logistically challenging or ethically difficult to explore experimentally. Additionally, modelling provides a quantitative link between formulation properties and pharmacodynamic outcomes, moving clinical decision-making beyond empirical trial-and-error approaches [26].

Nevertheless, several limitations remain. Many key parameters, including D_eff, P_SM and k_ads/k_des, originate from ex vivo or animal studies, and human-based measurements remain scarce. Furthermore, current models usually treat the biofilm as homogeneous, despite substantial structural and functional heterogeneity. More advanced numerical methods—including two- or three-dimensional diffusion–reaction models—may be required for applications where spatial gradients significantly influence transport. Finally, simulated exposure–effect relationships must be interpreted with caution, as pharmacodynamic modelling inherently extrapolates from concentration to outcome and can be sensitive to parameter uncertainty [26].

## 11. Conclusions

The mini-PBPK framework presented here provides an integrated, physiologically grounded approach to understanding local drug delivery in the oral cavity. By combining release kinetics, adsorption processes, biofilm diffusion, GCF-driven clearance and local PK/PD relationships, the model connects bench-scale experiments with in vivo exposure in a transparent and interpretable way.

This structure offers advantages for both researchers and clinicians. It provides a systematic method for analyzing how different formulations behave under varying oral conditions, and it supports more deliberate choices about carrier type, dosing strategy and expected duration of action. The framework also highlights how disease-related factors—particularly inflammation-driven increases in GCF flow—alter therapeutic profiles.

Future development may extend the model through patient-specific parameterization, imaging-derived geometries and machine-learning–assisted estimation methods. Incorporating multidimensional biofilm models or dynamic changes in mucosal permeability may further enhance predictive power. Ultimately, this approach serves as a bridge between quantitative pharmacology, dental biomaterials and clinical periodontology, promoting the design of more effective and personalized local therapies.

## Data Availability

No new experimental data were produced in this study. Model formulations, equations and illustrative simulations are available from the corresponding authors upon request.

## Abbreviations

The following abbreviations are used in this manuscript:

ACAT	Advanced Compartmental Absorption and Transit
BOP	Bleeding on Probing
CAL	Clinical Attachment Level
GCF	Gingival Crevicular Fluid
NLME	Nonlinear Mixed-Effects
OCCAT	Oral Cavity Compartmental Absorption and Transit
PBPK	Physiologically Based Pharmacokinetics
PD	Pharmacodynamics
PK	Pharmacokinetics
PPD	Probing Pocket Depth
SRP	Scaling and Root Planing
TMDD	Target-Mediated Drug Disposition
